# Professor Guiseppe Gozzetti

**DOI:** 10.1155/1996/28639

**Published:** 1996

**Authors:** Gian Luca Grazi

**Affiliations:** Second Department of Surgery University of Bologna Italy


### Professor Guiseppe Gozzetti (1935–1995)

On 10th June 1995, Professor Guiseppe Gozzetti, a master of Surgery, passed away. His humanity still remains in his fellows, colleagues and most of all, his patients, who are the witnesses of his *ars chirurgica* and human greatness.

Guiseppe Gozzetti was born on 1st Februray 1935 in Isola della Scala (VR), Italy. He graduated from the University of Bologna School of Medicine in 1960. He was one of the pioneers in Bologna in cardiovascular surgery, for which he had spent one year in Paris. The surgical school of Bologna owes its good reputation to his dedicated work as a researcher and as a surgeon. His career proceeded further when, in 1972, he was appointed director of the Surgical Department in Chieti where he stayed from 1972 to 1981. During that period the surgical department of Chieti, until then a local surgical ward, became one of the most important Italian surgical centres. Meanwhile, his interest grew toward hepatic surgery, of which he was to become, in the following decade in Bologna, an Italian and international leader. In 1981, he was called by the Faculty of Bologna and appointed director of the Clinica Chirurgica. From 1981 on, the career of Gozzetti become an endless sequence of successes and acknowledgements which has made him one of the European masters of surgery. He was one of the founders of the World Association of Hepato-Pancreato-Biliary Surgery (WAHPBS) which later became the International Hepato-Pancreato-Biliary Association (IHPBA). He was one of the current vice-chairman finally had the responsibility for organising the Second World Congress of the Association, which will be held in Bologna in June 1996 and will be now dedicated to his memory.

His surgical activity was wide-ranging throughout abdominal and thoracic surgical fields.

There are two main fields in which he achieved the most outstanding acknowledgements. The first was hepato-biliary surgery, where he has been author of over 600 publications and 5 books. It can be stated that there has not been any Italian or European congress on hepatic surgery in the last 10 years where Professor Gozzetti was not an invited speaker. A natural result of this activity is the establishment in Bologna of one of the most important European Liver Transplant Centres, to the financing and improvement of which he dedicated his time and energy till the last days of this life. It is due to his passionate work that many patients could stop travelling abroad in a "journey of hope", finding in Bologna an answer to their need for a transplant. About 200 liver transplantations were performed since May 1986 under his supervision. He personally performed the vast majority of them.

In addition, he stimulated the further development and activity of the ongoing kidney transplant programme in his Department: some 600 kidney transplantations have been performed, with more than 80 from living related donors.

The second field of interest for his clinical and research activity was the surgical treatment of Inflammatory Bowel Disease. In particular, he was involved in the surgical treatment of ulcerative colitis. He had the largest series in Italy of ileo-anal reservoirs. In 1989, he was the promoter and organiser of the first World Congress on ileo-anal pouches together with the Mayo Clinic and the Cleveland Clinic.

He recently joined the Editorial Boards of the journals *Transplant International* and *Liver Transplantation and Surgery*. Furthermore, he was co-editor of the surgical section of the journal *Hepatogastroenterology* and member of the editorial board of *HPB Surgery*.

For the last 8 consecutive years, he has been invited to the American College of Surgeons, of which he was a fellow, to present films of his operating techniques.

But for many years to come the most outstanding imprint that Professor Gozzetti leaves us will be as a man. Disease and pain have always accompanied him for the last 10 years. Nevertheless his patients have never lacked his time and attention, not even for a minute. On the contrary he used to spend much time talking and explaining to patients because this was his way of being a physician. Most of all his patients weep over him while the follows remember him as a master of surgery and a brave man.

Gian Luca Grazi MD
Second Department of Surgery
University of Bologna
November 1995

